# Circular RNA CircCCNB1 sponges micro RNA-449a to inhibit cellular senescence by targeting CCNE2

**DOI:** 10.18632/aging.102449

**Published:** 2019-11-25

**Authors:** Ai Qing Yu, Zhi Xiao Wang, Wu Wu, Ke Yu Chen, Shi Rong Yan, Ze Bin Mao

**Affiliations:** 1 Peking University Research Center on Aging, Department of Biochemistry and Biophysics, School of Basic Medical Sciences, Peking University Health Science Center, Beijing Key Laboratory of Protein Posttranslational Modifications and Cell Function, Beijing 100191, China; 2 Department of Cardiology, Taihe Hospital, Hubei University of Medicine, Shiyan, Hubei 442000, China; 3 Department of Immunology, School of Basic Medical Science, Tianjing Medical University, Tianjing 300070, China; 4 School of Pharmaceutical Sciences, Hubei Key Laboratory of Wudang Local Chinese Medicine Research, Hubei University of Medicine, Shiyan 442000, China

**Keywords:** senescence-associated circRNAs, CircCCNB1, miR-449a, cellular senescence, aging

## Abstract

Circular RNAs (CircRNAs) are a novel subset of non-coding RNA widely present in eukaryotes that play a central role in physiological and pathological conditions. Accumulating evidence has indicated that CircRNAs participated in modulating tumorigenesis by acting as a competing endogenous RNA (CeRNA). However, the roles and functions of CircRNAs in cellular senescence and aging of organisms remain largely obscure. We performed whole transcriptome sequencing to compare the expression patterns of circular RNAs in young and prematurely senescent human diploid fibroblast 2BS cells, and identified senescence-associated circRNAs (SAC-RNAs). Among these SAC-RNAs, we observed the significantly downregulated expression of CircRNAs originating from exons 6 and 7 circularization of the cyclin B1 gene (*CCNB1*), termed CircCCNB1. Reduced CircCCNB1 expression triggered senescence in young 2BS cells, as measured by increased senescence associated-beta-galactosidase (SA-β-gal) activity, enhanced expression of cyclin-dependent kinase inhibitor 1A (CDKN1A)/P21 and tumor protein 53 (TP53) expression, and reduced cell proliferation. Mechanistically, reduced CircCCNB1 level inhibited cyclin E2 (CCNE2) expression by modulating micro RNA (miR)-449a activity, which repressed cellular proliferation. Our data suggested that CircCCNB1may serve as a sponge against miR-449a to delay cellular senescence by targeting CCNE2. Targeting CircCCNB1 may represent a promising strategy for aging and age-related disease interventions. Furthermore, we also identified and characterized several kinds of the CircCCNB1-binding proteins (CBPs), which may contribute to the degradation of CircCCNB1.

## INTRODUCTION

Cellular senescence is a permanent and irreversible growth arrest, which is divided into replicative cellular senescence or premature cellular senescence [1]. After completing a limited number of divisions, eukaryotic cells retain their basic metabolism, but lose the ability to synthesize DNA, leading to the exhaustion of their proliferative potential and telomere erosion, a phenomenon termed ‘replicative senescence’ [2]. External stress can also induce cellular senescence called ‘premature senescence’, which is independent of telomere erosion, such stress including oncogene overexpression, ionizing radiation, oxidative stress, etc. [3]. Cellular senescence is mainly measured by (I) irreversible growth arrest; (II) altered cellular morphology; (III) senescence-associated β-galactosidase (SA-β-gal) activity; (IV) senescence-associated heterochromatin foci (SAHF); (V) senescence-associated secretory phenotype (SASP), etc. [4]. Mechanistically, P53-P21^CIP1^ and P16^INK4A^-Rb comprise of the major signaling pathway regulating cellular senescence. P53 and P16^INK4A^, therefore, are considered crucial regulatory factors for the induction of cellular senescence [5].

Cellular senescence is involved in modulating aging and aging-associated pathologies via the senescence-associated secretory phenotype (SASP) [6]. Growing evidence has implicated the accumulation of senescent cells are implicated in chronologic aging of organisms [7]. Several lines of evidence have suggested that cellular senescence is closely associated with age-related diseases, including osteoarthritis, atherosclerosis, cancer, Parkinson’s disease, Alzheimer’s and type 2 diabetes [8–13]. Local clearance of senescent cells by senolytic agents attenuates the development of aging-related diseases, including osteoarthritis and atherosclerotic [14–16]. Therefore, characterizing the regulatory mechanisms of cellular senescence may allow us to intervene in aging-related diseases.

MicroRNAs (miRNAs) are approximately 22-nucleotide, long and evolutionarily conserved noncoding (nc)RNAs that participate in the formation of the RNA-induced silencing complex (RISC) [17, 18]. miRNAs-RISC complexes modulate target gene expression by the interplay of miRNAs with their target mRNAs via partial sequence complementarity, leading to degradation and(or) reduced translation of target mRNAs [19–21]. By affecting target gene expression pattern, miRNAs play crucial roles in cellular processes, including proliferation, survival, differentiation and development [22]. Indeed, increasing evidence suggests that miRNAs are vital for modulating tumorigenesis and progression of various cancers [23–25]. However, the interaction between miRNAs and circRNAs in regulating cellular senescence is poorly understood.

Circular RNAs (CircRNA) generated by back-splicing are highly conserved and stable long non-coding RNAs abundant in eukaryotic transcriptomes [26]. CircRNAs are characterized by a stable loop structure with neither 5′ to 3′ polarity nor a polyadenylated tail [27]. High-throughput sequencing and novel computational approaches have identified increasing numbers of CircRNAs in various cell lines and species. Currently, the functions of most CircRNAs remain largely unexplored; however, the known functions include (I) sequestration of microRNAs or proteins [28]; (II) modulation of transcription and splicing [26]; (III) peptide or protein encoding [29–31]. CircRNAs are also involved in various pathological and physiological processes, including cancer development [32, 33], cardiovascular disease [34, 35], and aging [36]. Chu Xiao Liu and colleagues first reported that reduced CircRNA expression is closely related to systemic lupus erythematosus (SLE) [37]. However, molecular mechanisms and functions of CircRNAs in cellular senescence and aging of organisms remain largely unknown.

The present study identified senescence-associated CircRNAs (SAC-RNAs) by the whole-transcriptome sequencing, and revealed that CircCCNB1 is dramatically downregulated in replicative and prematurely senescent 2BS fibroblasts. Short hairpin RNA (shRNA)-induced knockdown of circular cyclin B1 (CircCCNB1) led to senescence in proliferating 2BS fibroblasts. Mechanistically, CircCCNB1 regulated cyclin E2(CCNE2) by controlling microRNA 449a (miR-449a) activity. Our data implicated that CircCCNB1-miR-449a-CCNE2 axis in the regulation of cellular senescence. Modulating miRNA activity by targeting SAC-RNAs can influence target protein expression, which may represent a promising strategy for aging and age-related disease interventions.

## RESULTS

### Identification of SAC-RNAs

**Figure 1:**
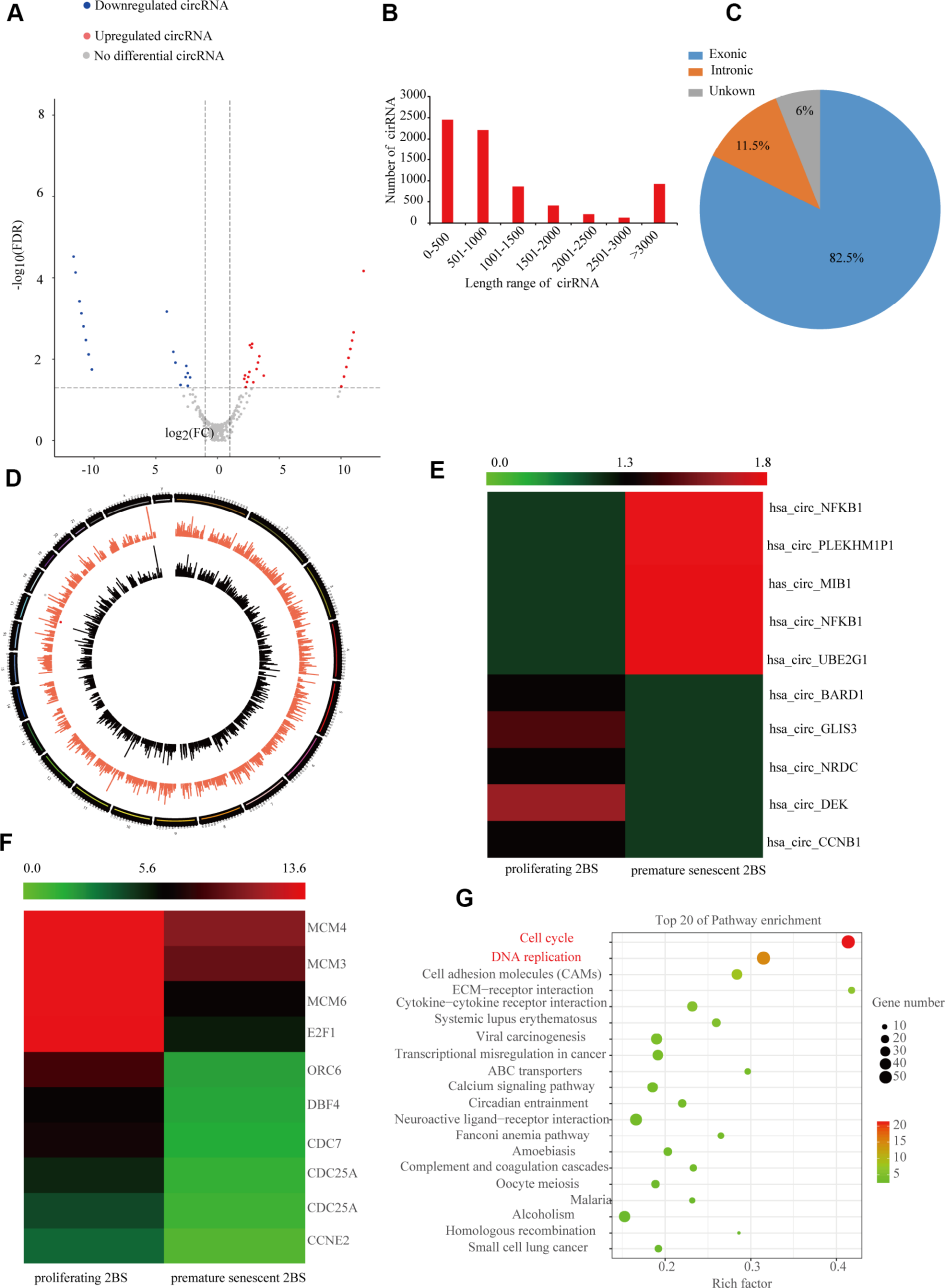
**Expression profile of SAC-RNAs and mRNA in proliferating and prematurely senescent 2BS fibroblasts.** (**A**) Volcano plot showing CircRNA expression in young and prematurely senescent 2BS fibroblasts. The red and green dots represent CircRNAs with statistically significant differences in expression. (**B**) Length distributions of the identified CircRNAs by RNA-Seq. x-axis: length of CircRNAs detected in this study. y-axis: number of CircRNAs classified according to length. (**C**) Pie chart showing the percentage of CircRNAs derived from different genomic regions. (**D**) Distributions of identified CircRNAs along the chromosomes. (**E** and **F**) Cluster heatmaps showing the five most increased and decreased CircRNAs and representative differentially expressed mRNAs. Each column indicates a sample and each row indicates an individual CircRNA or mRNA. (**G**) Kyoto Encyclopedia of Genes and Genomes (KEGG) of differentially expressed mRNAs.

To identify the profile of SAC-RNAs in 2BS fibroblasts, total RNA was isolated from young (proliferating) and irradiation-induced prematurely senescent 2BS fibroblasts. CircRNAs were identified by RNA-Seq, as described in the Materials and Methods part. With a cut-off criterion of log2FC > 2.0 and P < 0.05, we identified 143 CircRNAs that were differentially expressed between young and prematurely senescent 2BS fibroblasts, among which 87 were upregulated and 56 were downregulated (Figure 1A). Most of these CircRNAs originated from protein coding exons, while others aligned with introns or unknown sequences (Figure 1B). Most of the identified CircRNAs were less than 2000 nt (Figure 1C). The chromosome distribution of the CircRNAs showed no obvious differences between the young and premature senescent groups (Figure 1D). The five most highly increased and decreased CircRNAs are depicted in a heatmap (Figure 1E and a Suplementary Table 2). CircCCNB1(hsa_circ_0001495) is one of the most highly downregulated (0.00054- fold) CircRNAs. Moreover, using the same cut-off criteria, we also identified 1,719 mRNAs with differential expression, including 1,278 upregulated and 441 downregulated mRNAs; 10 representative differentially expressed mRNAs are shown in the heatmap in Figure 1F. Kyoto Encyclopedia of Genes and Genomes (KEGG) enrichment analysis showed that the cell cycle was the most significantly enriched pathway (Figure 1G). CCNE1 was the most downregulated (0.179-folds) cell cycle-related gene observed in the prematurely senescent 2BS fibroblasts. As cell cycle progression is a crucial characteristic of cellular senescence, we focused our attention on CircCCNB1 and CCNE2 in cellular senescence. The primary data of the RNA-Seq was provided in Supplementary dataset.

**Figure 2:**
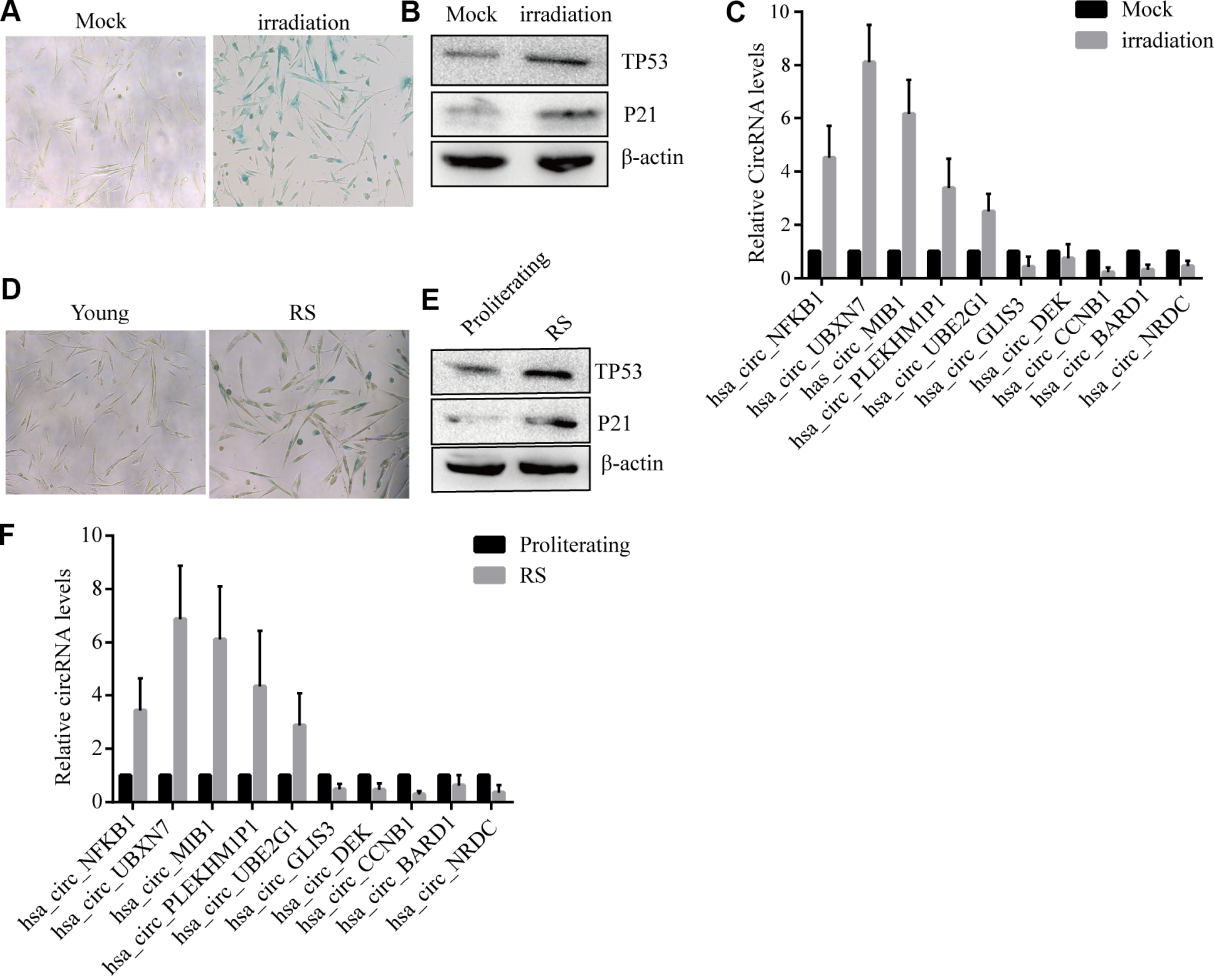
**Confirmation of SAC-RNAs expression profiles in prematurely senescent and RS 2BS fibroblasts.** (**A**) Senescence-associated beta-galactosidase (SA-β-gal) staining of in mock-treated (proliferating) and irradiation-induced (prematurely senescent) 2BS cells, bars, 100 μL. (**B**) Immunoblots analysis of P53 and P21levels in mock-treated and irradiation-induced premature senescent 2BS. (**C**) Quantitative reverse transcription-polymerase chain reaction (qRT-PCR) analysis of alterations of CircRNA expression in mock-treated and irradiation-induced 2BS cells. (**D**) SA-β-gal staining of proliferating and RS 2BS cells, bars, 100 μL. (**E**) immunoblots analysis of the levels of P53 and P21 in proliferating and RS 2BS cells. (**F**) qRT-PCR analysis of changes in CircRNAs expression in proliferating and RS 2BS cells.

The10 most highly increased and decreased CircRNAs identified by RNASeq of proliferating and prematurely and replicative senescent 2BS fibroblasts by qRT-PCR using specific divergent primers (provided in Suplementary Table 1). As shown, irradiation -induced premature and reduplicate senescent (RS) 2BS displayed a flattened and enlarged morphology and increased SA-β-gal activity (Figure 2A–2D). Western blot analysis showed increased levels of senescent markers such as CDKN1A*/*P21 andTP53 in premature and RS 2BS fibroblasts (Figure 2B–2E). qRT-PCR also showed the CircRNA expression pattern is to be appropriately consistent with RNASeq results, and that CircCCNB1 expression was dramatically downregulated in premature and RS 2BS fibroblasts. (Figure 2C–2F).

### Validation of CircCCNB1 and novel circular RNAs in human diploid fibroblasts

**Figure 3:**
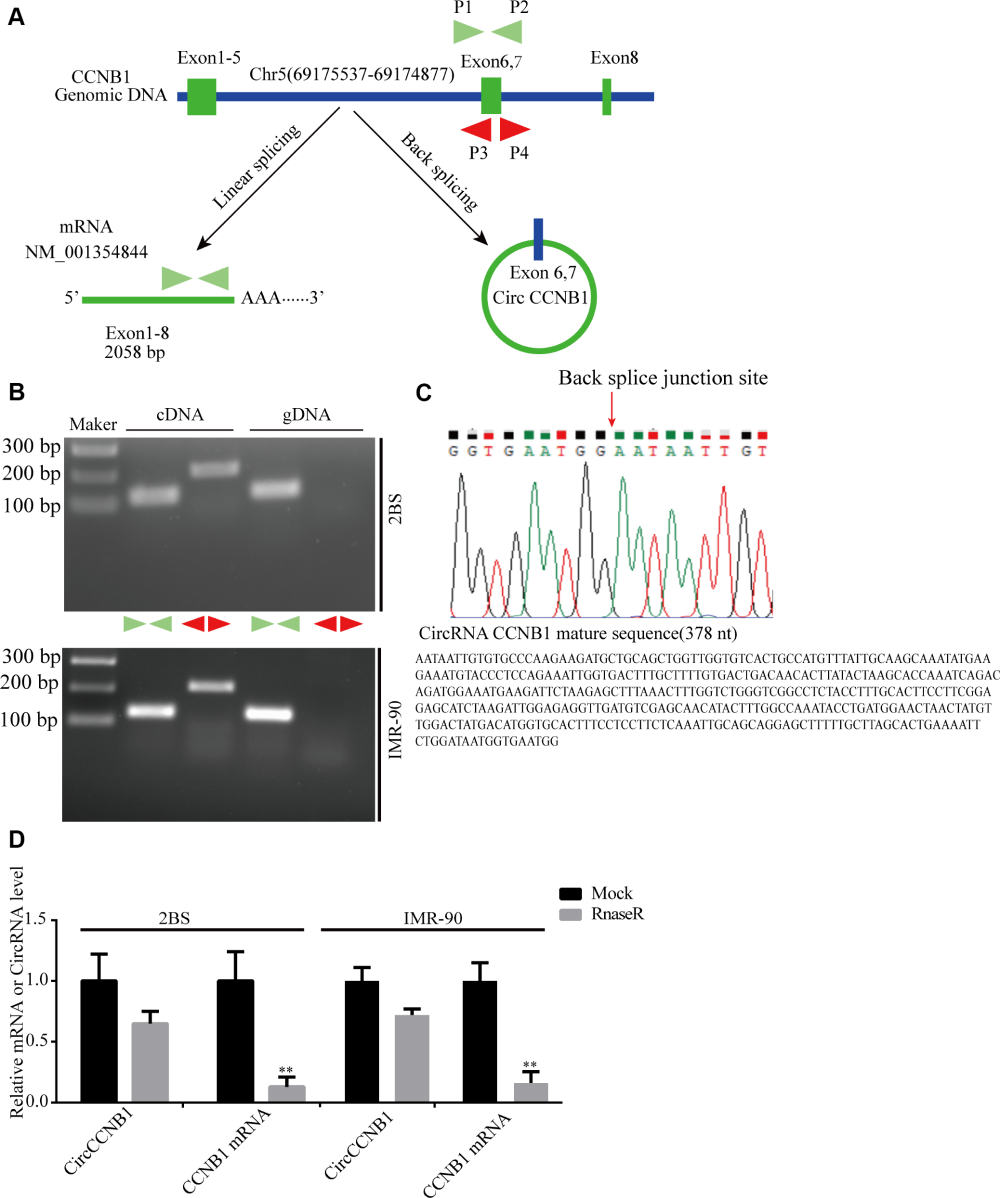
**Validation and annotation of CircCCNB1 and novel circular RNAs in human diploid fibroblasts.** (**A**) Illustration of the annotated genomic region of CCNB1, the putative different RNA splicing forms, and the validation strategy for circular exons 6 and 7 (CircCCNB1). Convergent (blue) and divergent (red) primers were designed to amplify the linear or back-splicing products. (**B**) Total RNA or genomic DNA isolated from 2BS or IMR-90 cells were subjected to polymerase chain reaction (PCR). (**C**) Upper panel: Red arrow showing the back-splicing of CCNB1 exons 6 and 7 confirmed by Sanger sequencing. Lower panel: CircCCNB1 mature sequence with 378 nt. (**D**) Total RNA from 2BS or IMR-90 cells with or without RNase-R treatment were subjected to qRT-PCR.

According to the RNASeq annotation of and NCBI alignment, CircCCNB1is a circular RNA and originating from exon6 and 7 circularization of the CCNB1 gene located at Chr5(69175537-69174877) (Figure 3A). To validate CircCCNB1 as an authentic circular RNA in human diploid fibroblasts, convergent and divergent primers were designed [38]. Using cDNA and genomic DNA from young 2BS and IMR-90 fibroblasts as templates, CircCCNB1 was only amplified by divergent primers in cDNA. We did not detect any amplification product in genomic DNA (Figure 3B). Sanger sequencing of the amplification product showed that CircCCNB1 is a true circular RNA with “head-to-tail” splicing sites and a 378 nt mature sequence (Figure 3C). Moreover, qRT-PCR confirmed that the CircCCNB1 was resistant to RNase R compared to RNase R-treated CCNB1 mRNA (Figure 3D). Based on current circbase annotation, we identified four novel circular RNAs from 10 most highly increased and decreased CircRNAs through RT-PCR Sanger sequencing (Supplementary Figure 1).

### CircCCNB1 plays a role in suppressing cellular senescence in human diploid fibroblasts

**Figure 4:**
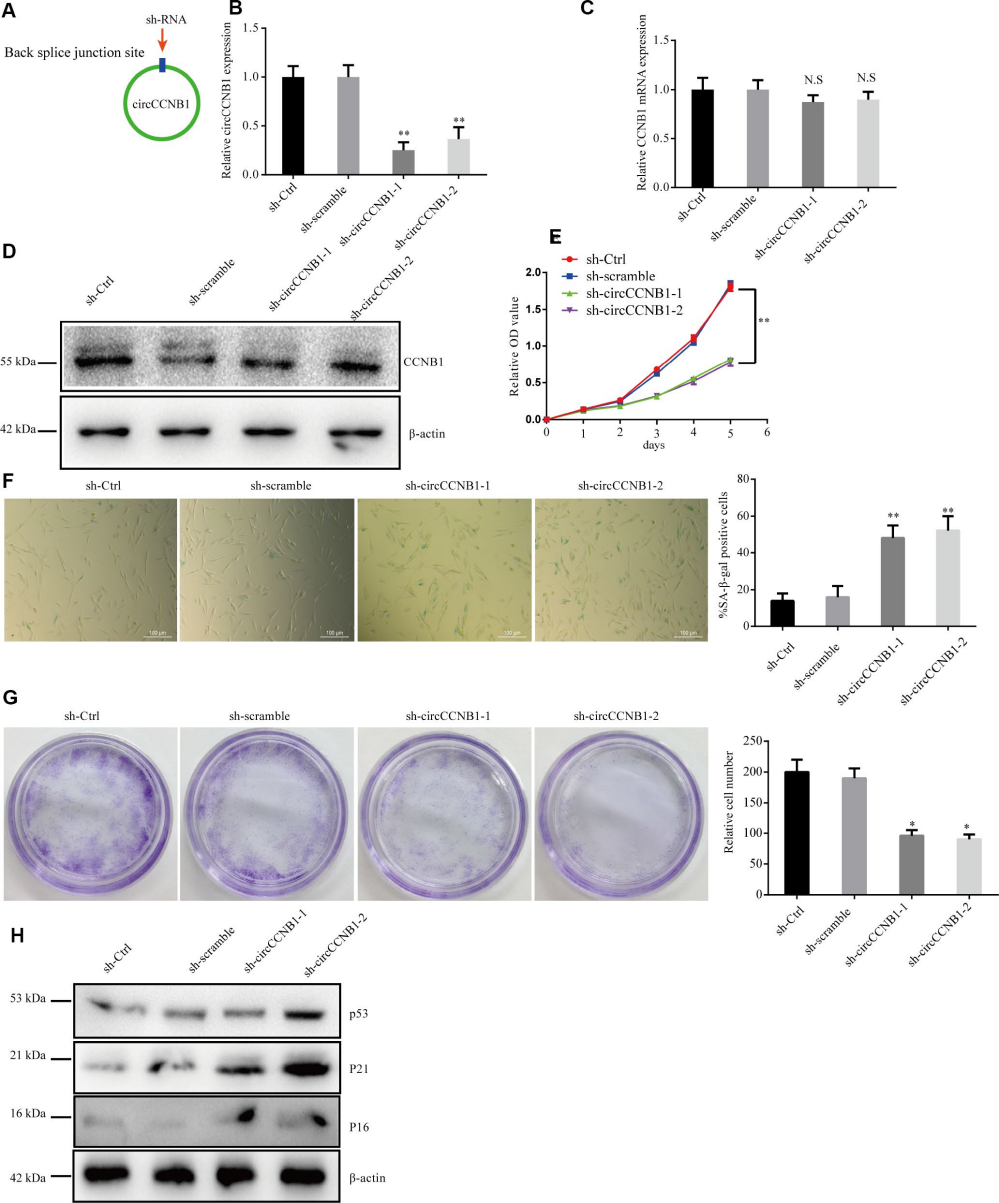
**CircCCNB1 suppresses cellular senescence in human diploid fibroblasts.** (**A**) Model showing shRNA specifically targeting the back-splicing sites of CircCCNB1. (**B**) qRT-PCR measuring the knock down efficiency by sh-CircCCNB1 in 2BS cells after transfection with lentiviruses expressing sh-ctr, sh-scramble, sh-circCCNB1-1, sh-circCCNB1-2, **P<0.01. (**C**) qRT-PCR detects CCNB1 mRNA level in 2BS cells transfected with lentiviral expressing sh-ctr, sh-scramble, sh-CircCCNB1-1and sh-CircCCNB1-2, N.S, nonsignificant. (**D**) Immunoblot analysis of CCNB1 protein level in 2BS cells transduced with lentiviruses expressing sh-ctr, sh-scramble, sh-CircCCNB1-1and sh-CircCCNB1-2. (**E**) CCK-8 measurement of the proliferative ability of 2BS cells after transfection with lentiviruses expressing sh-ctr, sh-scramble, sh-CircCCNB1-1, sh-CircCCNB1-2, **P<0.01. (**F**) SA-β gal staining of 2BS cells transfected with lentiviruses expressing sh-ctr, sh-scramble, sh-CircCCNB1-1and sh-CircCCNB1-2, bar, 100 μm, **P<0.01. (**G**) Clonogenicity assay of 2BS cells transfected with lentiviruses expressing sh-ctr, sh-scramble, sh-CircCCNB1-1and sh-CircCCNB1-2, *P<0.05. (**H**) Immunoblots analysis of P53, P21 and p16 levles in 2BS cells transfected with lentiviruses expressing sh-ctr, sh-scramble, sh-CircCCNB1-1and sh-CircCCNB1-2.

To determine the effect of CircCCNB1 on cellular senescence, CircCCNB1was knocked down in young 2BS cells (population doublings 18) using two short hairpin RNAs (shRNAs) specifically targeting the back-splicing sites through lentiviral infection (Figure 4A). After transfection, the relative CircCCNB1 expression was measured by qRT-PCR, which indicated that sh-CircCCNB1-1 and sh-CircCCNB1-2 led to a more than 50% downregulation of CircCCNB1 expression (Figure 4B), and had no effect on the expression of the linear transcript (Figure 4C, 4D). Reduced CircCCNB1 expression inhibited 2BS cells proliferation as measured by Cell Counting Kit-8 (CCK-8) assay (Figure 4E). CircCCNB1 knockdown also increased SA-β-gal activity (Figure 4F) and weakened the clonogenicity (Figure 4G) in 2BS cells. As the p53-p21 and p16-pRB pathways are the major classic pathways of cellular senescence, we detected these key protein levels in 2BS cells transfected with lentiviruses carrying sh-CircCCNB1. The data showed that CircCCNB1 knockdown increased p53, p21 and p16 expression (Figure 4H). Together, these data support the hypothesis that CircCCNB1 knockdown may induce the senescence phenotype in human diploid fibroblasts. CircCCNB1, therefore, may play a suppressive role in cellular senescence in human diploid fibroblasts.

### CircCCNB1 acts as a sponge of MiR-449a and suppresses miR-449a activity

**Figure 5:**
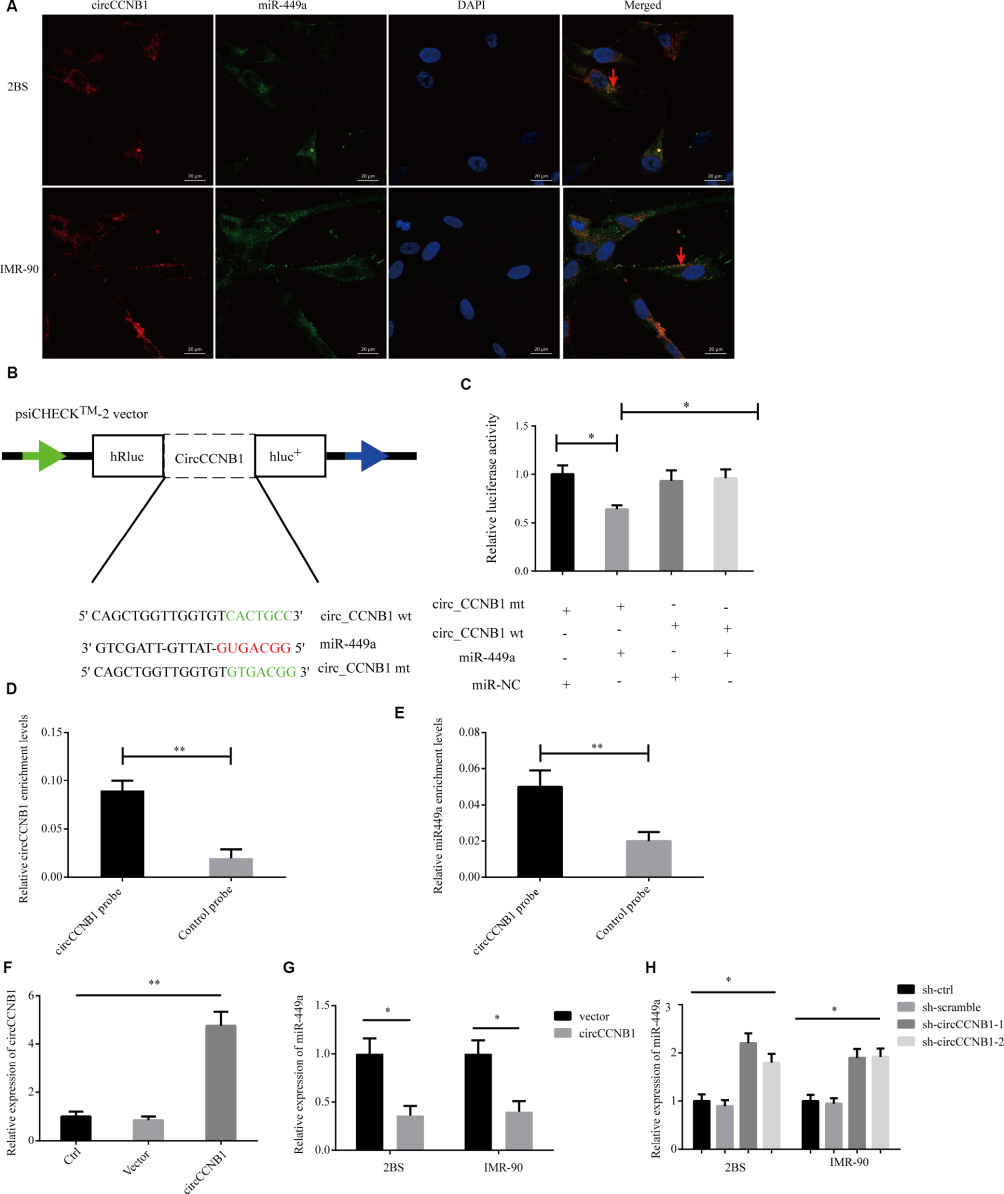
**CircCCNB1 functions as a miR-449a sponge and suppresses miR-449a activity.** (**A**) FISH was performed to observe for localization of CircCCNB1 (red) and miR-449a (green) in 2BS or IMR-90 cells. Scale bar, 20μm, red arrowhead indicates co-localization. (**B**) Schematic illustration of the CircCCNB1-wild-type (wt) and CircCCNB1-mutant (mut) dual luciferase reporter vectors. (**C**) Relative luciferase activities were measured in 293T cells after transfection with CircCCNB1-wt or CircCCNB1-mut and miR-449a mimics or miR-NC, respectively, *P<0.05. (**D** and **E**) RNA pull-down in 2BS cells, followed by qRT-PCR to detect the enrichment of CircCCNB1 and miR-449a, **P<0.01. (**F**) Overexpression of CircCCNB1 by qRT-PCR, **P<0.01. (**G**) Relative miR-449a expression in CircCCNB1-overexpressed 2BS or IMR-90 cells by qRT-PCR, *P<0.05. (**H**) Relative miR-449a expression in CircCCNB1 knockdown 2BS or IMR-90 cells by qRT-PCR. *P<0.05.

To characterize the molecular mechanism underlying CircCCNB1 function, we initially, predicted the potential binding sites of CircCCNB1 by miRNA response elements (MREs) using the TargetScan, miRanda database and RNAhybrid [39]. The intersection of TargetScan, miRanda and RNAhybrid showed that CircCCNB1 acted as a sponge for 13 miRNAs with 14 potential binding sites; among these, miR-449a possessed a conserved target site with a high score (Supplementary Figure 2). As previous investigations indicated that CircRNAs could serve as miRNA sponges in the cytoplasm [40], we conducted FISH assay in the human diploid 2BS fibroblasts and IMR-90 cells to observe the subcellular localization of CircCCNB1 and miR-449a. The results showed that circAGFG1 (red) and miR-449a (green) were predominately co-localized in the cytoplasm (Figure 5A). To demonstrate the bioinformatics prediction, we conducted a dual-luciferase reporter assay was conducted in 293T cells. The full-length of CircCCNB1-wt and mutant without miR-449a binding sites were subcloned into the psiCHECK2 luciferase reporter vector (Figure 5B). The results showed that miR-449a mimics could significantly reduce the luciferase activity of CircCCNB1-wt but not the mutant and miR-NC groups, which suggested a direct interaction between CircCCNB1and miR-449a (Figure 5C). To further validate the interaction of CircCCNB1and miR-449a in 2BS cells, we performed a CircRNA pull-down assay with specific biotin-labeled CircCCNB1 probes. Specific enrichment of CircCCNB1 and miR-449a was detected by qRT-PCR in the CircCCNB1 probe group compared to that for the control probe (Figure 5D, 5E). In addition, CircCCNB1 overexpression led to a dramatic decrease, while circCCNB1 knockdown results to significantly increase the expression of miR-449a in 2BS and IMR-90 cells (Figure 5F–5H).

Together, these data demonstrated that CircCCNB1 could act as a sponge for miR-449a in human diploid fibroblasts.

### CCNE2 is directly regulated by miR-449a and indirectly regulated by CircCCNB1

**Figure 6:**
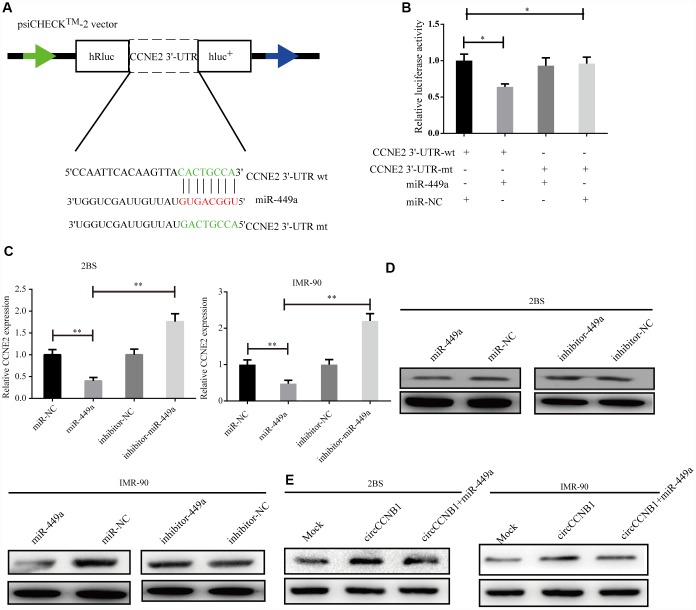
**CCNE2 is directly regulated by miR-449a and indirectly regulated by CircCCNB1.** (**A**) Schematic illustration of CCNE2 3′-UTR wild-type (wt) and CCNE2 3′-UTR-mutant (mut) dual luciferase reporter vectors. (**B**) The relative luciferase activities were detected in 293T cells after transfection with CCNE2 3′-UTR wt and CCNE2 3′-UTR -mut and miR-449a mimics or miR-NC, respectively, *P<0.05. (**C** and **D**) Relative mRNA and protein levels of CCNE2 in 2BS or IMR-90 cells after transfection with miR-NC, miR-449a, inhibitor-NC and inhibitor-449a using qRT-PCR and western blot, respectively, **P<0.01.

According to TargetScan, miRanda and RNAhybrid prediction, both CircCCNB1 and CCNE2 3'-UTR wt could bind to miR-449a through the same MRE. To verify this prediction, we performed a dual luciferase reporter assay by subcloning subcloned the CCNE2 3'-UTR wt and mutant sequence with or without miR-449a binding sites into psiCHECK2 luciferase reporter vector, respectively (Figure 6A). The results showed significantly decreased luciferase activity for the reporter vector carrying the CCNE1 3’UTR-wt sequence by miR-449a mimics but not by mutant and miR-NC (Figure 6B). The miR-449a mimics significantly reduced the mRNA levels of CCNE2, while the miR-449a inhibitor dramatically increased the mRNA levels of CCNE2 in 2BS and IMR-90 cells (Figure 6C). Additionally, as shown in Figure 6D, the protein levels of CCNE2 were accordingly altered in 2BS and IMR-90 cells. To verify whether CircCCNB1 indirectly regulated CCNE2 expression, we found that CircCCNB1 knockdown markedly decreased CCNE2 expressions, while CircCCNB1 overexpression resulted in the opposite aspect. The increase or decrease of CCNE2 induced by CircCCNB1 overexpression or knockdown could be reversed by miR-449a mimics or inhibitors, respectively (Figure 6E). Collectively, these data indicated that CircCCNB1 modulated CCNE1 expression by acting as a CeRNA for miR-449a in human diploid fibroblasts.

### CircCCNB1 suppresses cellular senescence through sponging miR-449a to regulate CCNE2

**Figure 7:**
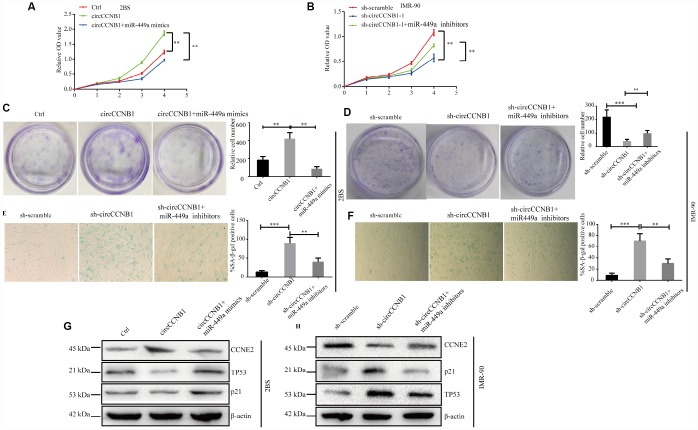
**CircCCNB1 suppresses cellular senescence by sponging miR-449a to regulate CCNE2.** (**A** and **B**) CCK-8 analysis was performed in 2BS or IMR-90 cells after transfection with ctrl, CircCCNB1 and CircCCNB1+miR-449a mimics, or sh-scramble, sh-CircCCNB1-1 and sh-CircCCNB1-1+miR-449a inhibitors, respectively, **P<0.01. (**C** and **D**) Clonogenicity assay of 2BS or IMR-90 cells after transfection with ctr, CircCCNB1 and CircCCNB1+miR-449a mimics, or sh-scramble, sh-CircCCNB1-1 and sh-CircCCNB1-1+miR-449a inhibitors, respectively, **P<0.01, ***P<0.001. (**E** and **F**) SA-β gal of 2BS or IMR-90 after transfection with sh-scramble, sh-CircCCNB1-1 and sh-CircCCNB1-1+miR-449a inhibitors respectively, bar, 100μm, **P<0.01, ***P<0.001. (**G** and **H**) Immunoblot analysis of CCNE2, P53 and P21 levels in 2BS or IMR-90 cells after transfection with ctr, CircCCNB1 and CircCCNB1+miR-449a mimics, or sh-scramble, sh-CircCCNB1-1 and sh-CircCCNB1-1+miR-449a inhibitors, respectively.

To determine whether CircCCNB1 inhibited cellular senescence through the CircCCNB1/miR-449a/CCNE2 axis, rescue experiments were performed using miR-449a mimics and inhibitors. The data indicated that miR-449a mimics reversed the proliferation and clonogenicity-promoting effects induced by CircCCNB1overexpression in human diploid fibroblasts, whereas miR-449a inhibitors could rescue proliferation and clonogenicity-suppressing effects induced by circCCNB1 knockdown (Figure 7A–7D). Furthermore, miR-449a inhibitors also could reverse the enhanced SA-β-gal activity mediated by knockdown of CircCCNB1 (Figure 7E, 7F). Except for this, the immunoblots demonstrated that CircCCNB1overexpression enhanced the protein levels of CCNE2, and reduced protein levels of p21 and p53, while knockdown of CircCCNB1led to the opposite results. The effects caused by CircCCNB1 overexpressing or silencing were reversed by miR-449a mimics or inhibitors, respectively (Figure 7G, 7H). These data indicated that CircCCNB1 might act as a CeRNA for miR-449a to regulate CCNE2 expression to inhibit cellular senescence of human diploid fibroblasts.

### Identification of CircCCNB1-bound proteins (CBPs) profile

**Figure 8:**
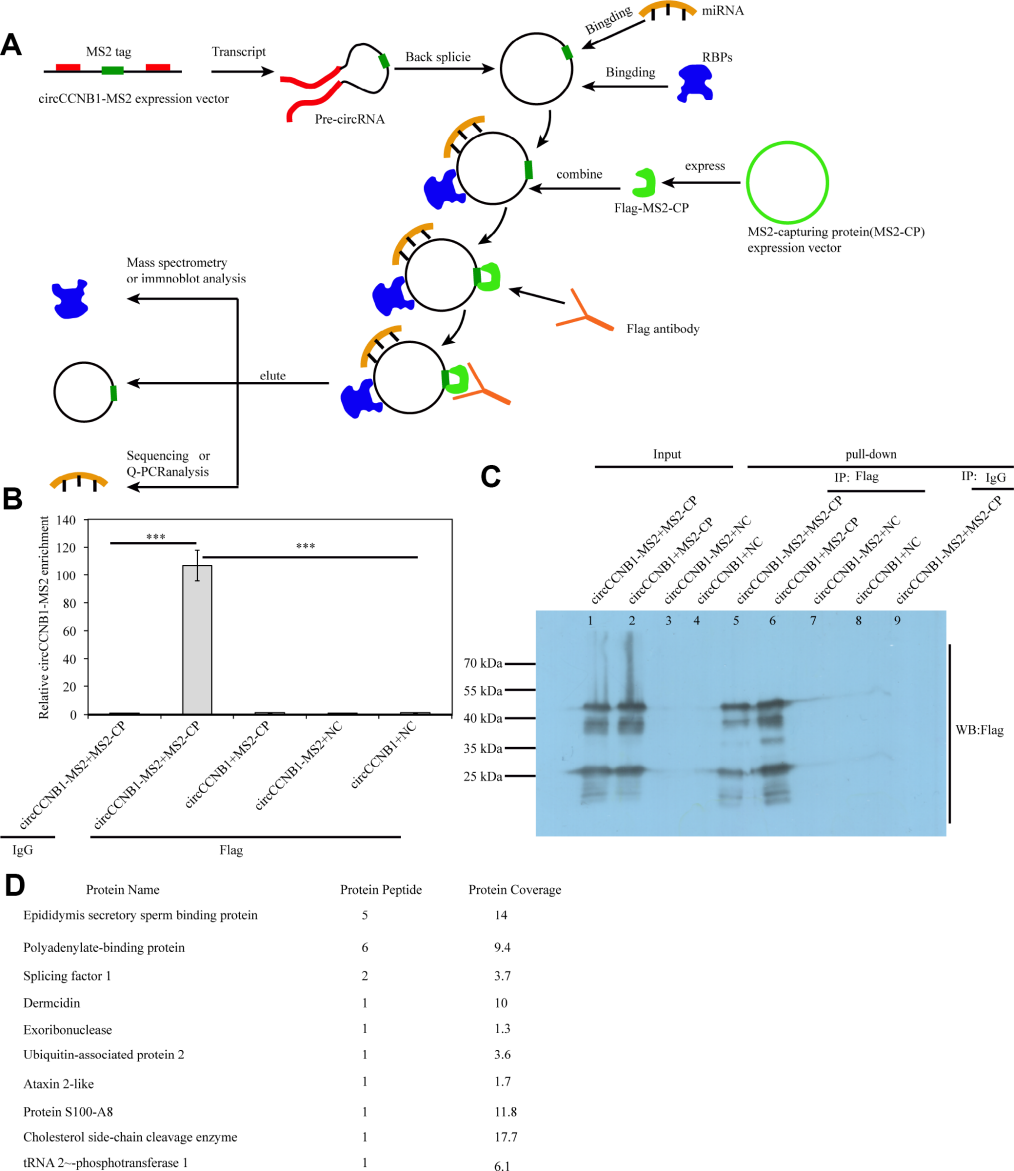
**Identification and characterization of CircCCNB1-binding proteins (CBPs).** (**A**) Schematic illustration of the modified RNA pulldown. Briefly, an MS2-tagged RNA was introduced into a CircRNA to express this specific tag(CircRNA-MS2) in cells; A Flag-tagged MS2-capturing protein(flag-MS2-CP) was constructed to specifically interact with the MS2 tag to capture CircRNA-MS2; Immunoprecipitation of the flag-MS2-CP-CircRNA-MS2 complex was performed using the flag antibody to identify proteins or miRNA molecules that might interact with CircRNA. (**B**) qRT-PCR detected the relative enrichment of circRNA-MS2 in HEK293T after transfection with the indicated plasmids, ***P<0.001. (**C**) Immunoprecipitation (IP) and immunoblot identification of CBPs.

Since long intergenic non-coding (linc)RNAs have been reported to bind proteins [41], we performed an improved RNA pulldown method to identify potential CBPs (Figure 8A) by constructing expression vectors - introducing RNA-tagged MS2 into CircCCNB1 (circCCNB1-MS2) and MS2-capturing protein (MS2-CP). We first measured the CircCCNB1 and CircCCNB1-MS2 expression efficacy in HEK293T by qRT-PCR and immunofluorescence prior to performing the pulldown assay (Supplementary Figure 3A, 3B). The results indicated that both were successfully expressed in HEK293T cells. Next, we co-transfected CircCCNB1-MS2+MS2-CP, CircCCNB1+MS2-CP, CircCCNB1-MS2+NC and CircCCNB1+NC into HEK293T cells respectively. Forty-eight hours after transfection, cell lysates were collected for pulldown analysis. Immunoprecipitats from cell lysate using anti-flag or immunoglobulin G IgG was subjected to qRT-PCR and immunoprecipitation (IP) analysis. qPCR analysis showed and that significant enrichment of CircCCNB1-MS2 in flag-enriched immunoprecipitats compared to IgG (Figure 8B). IP and immunoblot analysis showed and validated successful enrichment of CBPs in the CircCCNB1-MS2+MS2-CP transfected group compared to those in the corresponding group (Figure 8C). Furthermore, we performed mass spectrometry analysis of the enrichment of CircCCNB1-MS2+MS2-CP transfected precipitants to characterize these CBPs. We identified 10 CBPs by comparing identified-protein types between the CircCCNB1-MS2+MS2-CP-transfected (Supplementary Table 3) and circCCNB1+MS2-CP-transfected group (Figure 8D, Supplementary Table 4).

## DISCUSSION

Cellular senescence plays an essential role in tissue homeostasis. Dysfunction of cellular senescence is intimately associated with senescence-associated human diseases [42]. However, the molecular mechanisms underlying these diseases remain largely unexplored. Therefore, the novel and robust molecular targets that modulate cellular senescence require further investigation. In recent years, a growing number of CircRNAs have been discovered in multiple tissues and cell lines, especially in tumor tissues and cancer cells, by deep sequencing technology. CircRNAs possess an obvious advantage over their linear counterparts as promising diagnostic markers or therapeutic targets for many diseases for their stability and resistance to RNase R digestion [43]. CircRNAs are typically present at low abundance and often function in a cell type- and tissue-specific manners [26]. Although most known CircRNAs have been well functionally characterized in tumor tissues and cancer cells, the biological function of most circRNA in senescent cells and aged tissues remains largely unknown.

This study used RNA-seq to determine the expression profiles of CircRNAs and mRNA in young and irradiation-induced senescent 2BS cells. Subsequently, we characterized four novel CircRNAs from the five most-highly increased and decreased SAC-RNAs. Among the SAC-RNAs, CircCCNB1 attracted our attention in bioinformatics analysis for its involvement in the regulation of CCNE2 expression. Currently, the biological functions of CircRNAs remain largely unknown. Here, we focus our attention on the biological functions of CircCCNB1 as a sponge to sequester microRNAs or proteins to modulate cellular senescence in human diploid fibroblasts.

### CircCCNB1-binding microRNAs in human diploid fibroblasts

Increasing evidence has indicated that myriad CircRNA can sequester microRNAs to modulate miRNA target genes gene expression in many physiological and pathological processes [44, 45]. Our bioinformatics analyses indicated that CircCCNB1 harbored MREs of miR-449a based on bioinformatics analyses. FISH experiments demonstrated co-localization of CircCCNB1 and miR-449a in the cytoplasm of human diploid fibroblasts. We, therefore, assumed that CircCCNB1 modulated cellular senescence by sequestration of miR-449a. Dual-luciferase reporter and RNA pulldown assays confirmed that CircCCNB1 directly interplayed with miR-449a in human diploid fibroblasts. Additionally, CircCCNB1ovexpression and knockdown resulted in the contrary expression changes in miR-449a by qRT-PCR. Collectively, these data suggested that CircCCNB1 could act as a sponge in human diploid fibroblasts.

CeRNA analyses showed that CircRNAs serve as a CeRNA to modulate miRNA target gene expression [46]. We found that CCNE2, a crucial cell cycle regulator, CircCCNB1 were co-downregulated in irradiation induced and prematurely senescent 2BS cells. Notably, KEGG pathways analyses showed the cell cycle to be the most significantly enriched pathway, which preliminarily indicated that CircCCNB1may modulate cellular senescence through acting as a CeRNA to indirectly regulate CCNE2. Therefore, we performed a bioinformatics analysis, which suggested that CCNE2 was a potential target of miR-449a based on the intersection of TargetScan, miRanda and RNAhybrid analysis. Subsequently, a dual-luciferase reporter assay confirmed that miR-449a could directly bind to the 3′-UTR of CCNE2. In addition, overexpression of miR-449a led to reduced CCNE2 expression, whereas downregulation of miR-449a showed a contrary effect, suggesting that CCNE2 is a target gene of miR-449a. To verify the crosstalk between CircCCNB1 and CCNE2, we observed that CircCCNB1 overexpression increased CCNE2 translation whereas knockdown of CircCCNB1 showed the opposite impact. Moreover, these effects could be partially ablated by miR-449a mimics or inhibitors, respectively. Thus, CircCCNB1 overexpression led to reduced p21 and p53 levels, and increased SA-β-gal activity, proliferative ability and clonogenicity in human diploid fibroblasts, whereas CircCCNB1 knockdown showed the opposite effects. Furthermore, these effects were partially abolished by miR-449a mimics or inhibitors, respectively. Collectively, these data support the notion that CircCCNB1 functions as a CeRNA to suppress cellular senescence by regulating the miR449a/CCNE2 axis. In several lines of cancer, CCNE2 expression is modulated by miRNA or long non-coding (lncRNA), which play a key role to influence tumorigenesis and progression. Feng et al. reported that KCNQ1OT1 functioned as an oncogene by regulating the miR-145/CCNE2 axis to attenuate breast cancer development [47]. Similarly, miR-3607-3p acted as a tumor suppressor by sponging CCNE2 to suppresses non-small cell lung cancer [48]. However, the regulatory role of CircCCNB1/miR449-a/CCNE2 in cancer requires further investigation.

### CircCCNB1- sequestrated proteins

A diverse catalog of CircRNAs can bind or sequestrates proteins, by which they can interact with different proteins to exert different biological function [41, 49, 50]. We developed a modified pulldown method to characterize the CBPs. Our data showed the successful pulldown and identification of several types of CircCCNB1-bound proteins. Among these, exoribonuclease and Ubiquitin-associated protein 2 caught our attention for their potential involvement in the degradation of circCCNB1 and circCCNB1-bound proteins.

**Figure 9:**
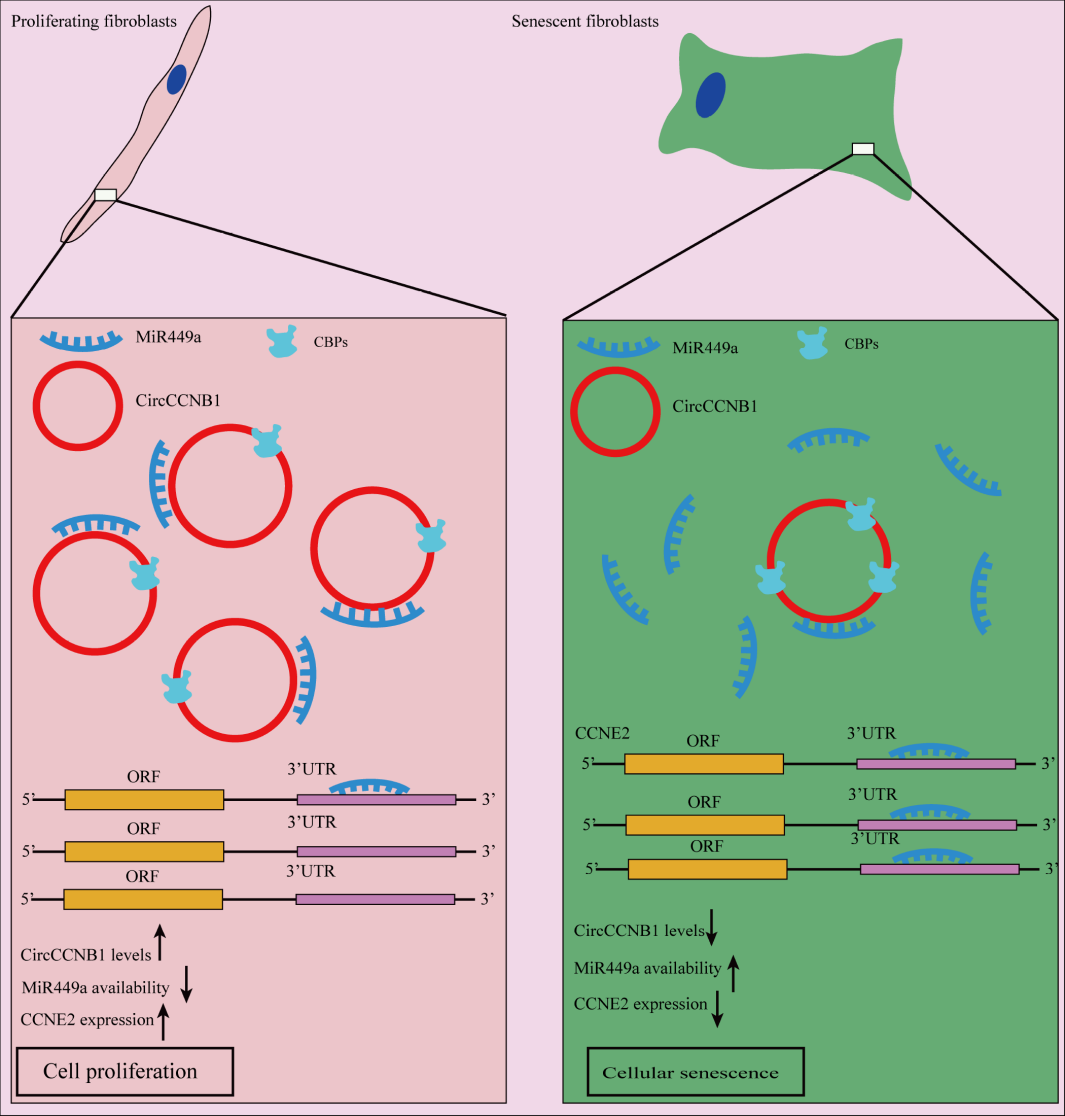
**Schematic diagram of CircCCNB1 function in human diploid fibroblasts.** Our model suggests that CircCCNB1 functions as a CeRNA to sponge miR-449a whereby CircCCNB1 facilitates the expression of the target *CCNE2* required for cell cycle development by preventing miR-449a from acting on the target mRNAs. The reduced expression of CircCCNB1 in senescent cells leads to elevated levels of functional miR449a, which in turn represses miR-449a target gene expression contributing to growth suppression and cellular senescence.

In summary, our model suggests that CircCCNB1 exerts its biological function through sequestrated microRNAs or proteins in human diploid fibroblasts. In CircRNA -sequestrated microRNAs aspect, we demonstrated that CircCCNB1 sponges miR-449a to inhibit cellular senescence by targeting CCNE2. Regarding CircRNA sequestrated proteins, we identified CBPs, which may be implicated in the degradation of CircCCNB1 and CircCCNB1-bound proteins (Figure 9).

## MATERIALS AND METHODS

### Cell lines and cell culture

Human diploid fibroblasts 2BS and IMR-90 cells were purchased from the National Institute of Biological Products (Beijing) China. HEK293 T cell lines were preserved by our lab. The cells were cultured in Dulbecco’s modified Eagle’s medium (Invitrogen, USA) supplemented with 10% fetal bovine serum (Invitrogen, USA), 100 U/mL penicillin and 100 mg/mL streptomycin. All cell lines were cultured at 37 °C with 5% CO _2_ in a humidified incubator.

### Immunoblots and antibodies

Whole cell lysates were prepared by incubating the cells in lysis buffer containing a protease inhibitor cocktail (Roche, Switzerland) and phosphatase inhibitor (Beyotime, China). The supernatants were subjected to separation by 10% sodium dodecyl sulfate-polyacrylamide gel electrophoresis (SDS-PAGE) and electrotransferred onto a polyvinylidene difluoride (PVDF) (Bio-Rad, USA). After blocking with 5% skimmed milk powder, the membranes were incubated with primary antibodies against CCNB1(no. ab72), P21(no. ab109520), CCNE2(no. ab32103), P16(no.ab189034) (1:1000 dilution, Abcam, Burlingame, CA, USA), P53(no. sc-126)(1;1000, *Santa Cruz* Biotechnology, USA) and β-actin(no.PM053) (1;1000 dilution, MBL, Japan) at 4 °C overnight. After washing three times, the membranes were then incubated with secondary antibodies (1:10000 dilution, EarthOx Life Sciences, USA) at room temperature for 1 h. Enhanced chemiluminescence (ECL kit, Millipore, USA) was used for visualization.

### The whole transcriptome sequencing

Total RNA extracted from the proliferating 2BS fibroblasts and irradiation-induced senescent 2BS fibroblasts. The whole transcriptome sequencing was conducted by Novel Bioinformatics Ltd., Co. In brief, the qualified RNA was used for cDNA Library Construction using the NEBNext® Ultra™ Directional RNA Library Prep Kit for Illumina according to the manufacturer’ s instructions. Generally, the protocol consists of the following steps: depletion of rRNA and fragmented into 150-200 bp using divalent cations at 94 °C for 8 min. The cleaved RNA fragments were reverse-transcribed into first-strand cDNA, second-strand cDNA synthesis. The cDNA library had been size selected by PAGE Gel electrophoresis for miRNA sequencing. After completing transcriptome sequencing, we performed a RNA sequencing Mapping to count mRNA and lncRNA counts and determine the gene expression. Finally, unmapped Reads was collected to identify and quantified the circRNAs.

### Vector construction and cell transfection

To knock down CircCCNB1, two shRNAs targeting the back-splice junction site of CircCCNB1 and a shRNA-scramble were synthesized. ShRNA against CircCCNB1 and a negative control shRNA-scramble (sh-CircCCNB1-1, sh-CircCCNB1-2, and sh-scramble) were synthesized and cloned into *pLent-U6-GFP-Puro vectors (Mailgene biosciences, Beijing, China),* named as sh-circCCNB1-1, sh-circCCNB1-2 and sh-scramble, respectively. All vectors were validated by Sanger sequencing. Cell transfections of sh-RNA were conducted by lentiviral infection. MiRNA mimics and inhibitors were purchased from (Sangon Biotech, Shanghai, China), and cell transfections were conducted using RFect^PM^ transfection reagent (Baidai biosciences, Changzhou, China). To overexpress CircCCNB1 and CircCCNB1-MS2, the full-length sequences of both were amplified in 293T cells and cloned into overexpression vector pLC5-ciR overexpression vector (Geneseed, Guangzhou, China), containing a front and back circular frame; a mock vector with no CircCCNB1 sequence served as a control, named plv-CircCCNB1 and plv-vector, respectively.

### qRT-PCR

Total RNA was exacted by Trizol reagent (Invitrogen) and was quantified using Nanodrop 2000 spectrophotometer (Thermo Scientific, MA, USA). Then 2μg RNA was reverse transcribed into cDNA using a Rever Tra Ace qPCR RT Kit (TOYOBO, Japan). qRT-PCR was conducted on a *7500* Real-Time PCR System (Thermo Fisher Scientific, MA, USA) using SYBR Green Realtime PCR Master Mix (TOYOBO, Japan). Glyceraldehyde 3-phosphate dehydrogenase (GAPDH) was used as an internal reference for quantification of CircRNA and mRNA, and U6 for miRNA. The specific primers are listed in Supplementary Table 1. The relative expression levels of CircRNA, mRNA and miRNA were calculated by the ^2 –ΔΔCT^ method.

### Luciferase reporter assay

The sequences of CircCCNB1 and CCNE2 targeted by miR-449a, and their corresponding mutant forms without miR-449a binding sites (circAGFG1-wild-type [wt], circAGFG1-mutant [mt], CCNE2, 3′UTR-wt, and CCNE1 3′UTR-mt) were subcloned into psiCHECK2 luciferase reporter vector (Promega, Madison, WI, USA). All constructed plasmids were validated by sequencing. The relative luciferase activity was measured using a Dual Luciferase Assay Kit (Promega, Madison, WI, USA) according to the manufacturer's directions.

### Fluorescence in situ hybridization (FISH)

FISH assays were performed to observe for localization of CircCCNB1 and miR-449a in 2BS and IMR-90 cells. In brief, cells were prehybridized at 55 °C for 2h before hybridization, with specific biotin-CY3-labeled CircCCNB1 (gcacacaattattccattcaccatt), and digoxin-FITC-labeled miR-449a probes (accagctaacaatacactgcca) (Geneseed, Guangzhou, China) at 37 °C overnight and finally dyed before 4′,6-diamidino-2-phenylindole (DAPI). Slides were photographed under a fluorescence microscope.

### RNA pull-down

RNA pull-down assays were conducted as described previously. Briefly, 2BS cells were lysed with lysis buffer and incubated with specific CircCCNB1 probes synthesized by Geneseed Biotech. The cell lysates were then incubated with streptavidin-coated magnetic beads to pull down the biotin-labeled RNA complex. After washing with the wash buffer, the RNA complexes bound to the beads were eluted and purified. The abundance of CircCCNB1 and miR-449ap were then analyzed with specific primers by qRT-PCR.

### Senescence-associated beta-galactosidase (SA-β-gal) staining

SA-β-gal activity was assessed as described previously [51].

### Cell counting Kit-8 (CCK-8) assay

Cells were seeded into 96-well plates at a density of 1×10^4^. Seeded cells were treated with of CCK-8 solution (Dojindo Laboratories, Japan). After incubation at 0, 24, 48, 72, 96 and 120 h, respectively, the absorbance of cells at each time point was analyzed at 450 nM using a microplate reader according to the manufacturer’s instructions.

### Colony formation assay

Cells were seeded into 3.5 cm petri dish plates, and incubated at 37°C for 14 days. Colonies were fixed in 4 % polyformaldehyde, stained with 0.1% crystal violet, imaged and counted.

### Statistics analysis

Means ± standard deviation (SD) were used to represent quantitative data from three independent biological repetitions. Statistical analyses were performed using IBM SPSS Statistics for Windows, version 25.0, and statistical significance between groups was determined by Student’s t -tests or analysis of variance (ANOVA). P<0.05 indicated statistically significant differences.

## Supplementary Material

Supplementary Figures

Supplementary Tables
