# Supplementary material for: Circular RNA CircCCNB1 sponges micro RNA-449a to inhibit cellular senescence by targeting CCNE2
Source: Aging (Albany NY). 2019 Nov 25;11(22):10220–41. doi: 10.18632/aging.102449 (PMC6914408; doi:10.18632/aging.102449)
Supplement: Supplementary Figures [file aging-11-102449-s002..pdf]

SUPPLEMENTARY FIGURES

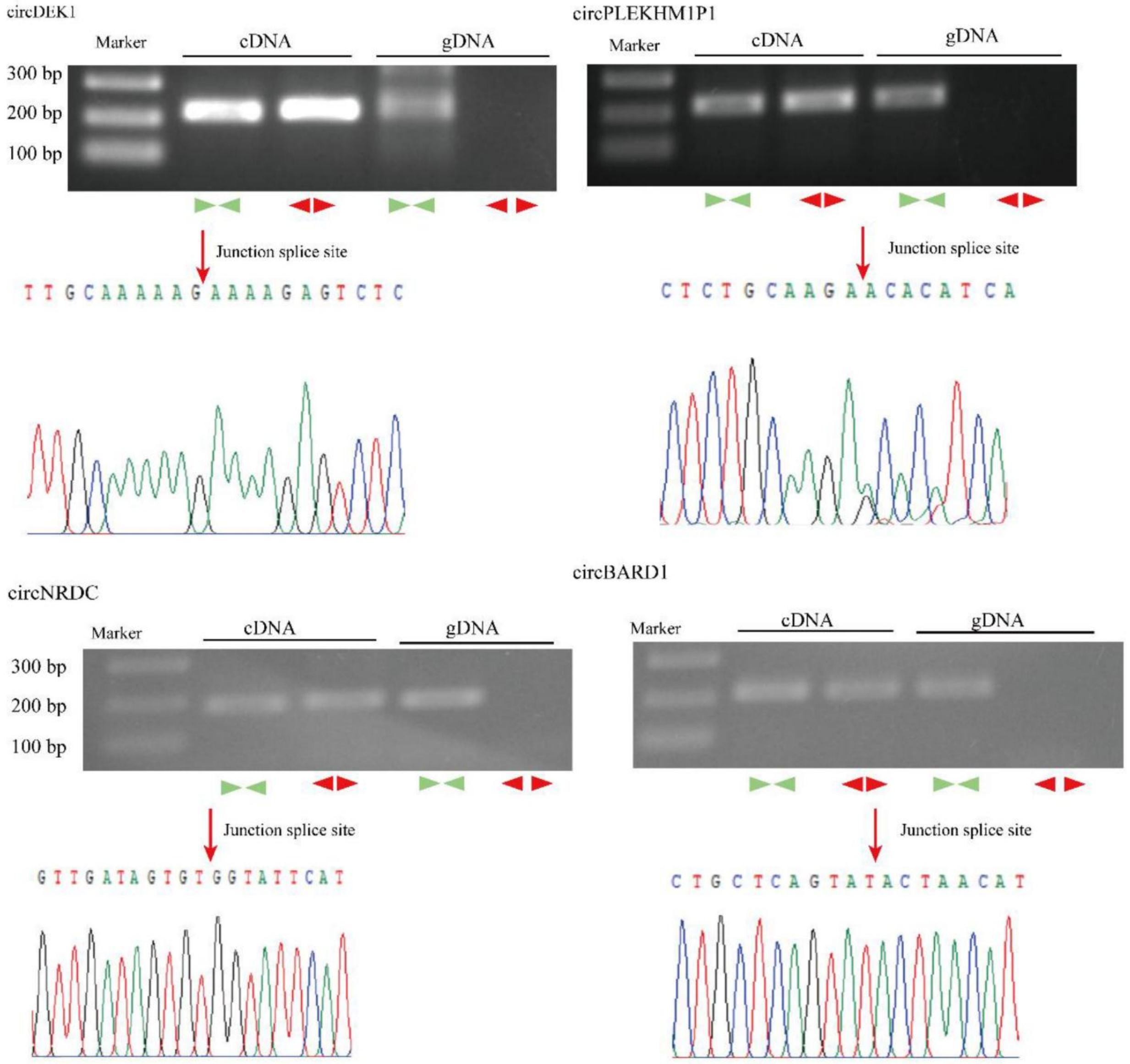

Supplementary Figure 1. Validation of four novel Circular RNAs from the 10 mostly increased and decreased CircRNAs by RT-PCR Sanger sequencing. Upper panel: RT-PCR products amplified using Convergent (blue) and divergent (red) primers under corresponding template; Lower panel: showing the back-spliced events of CircRNAs by Sanger sequencing.

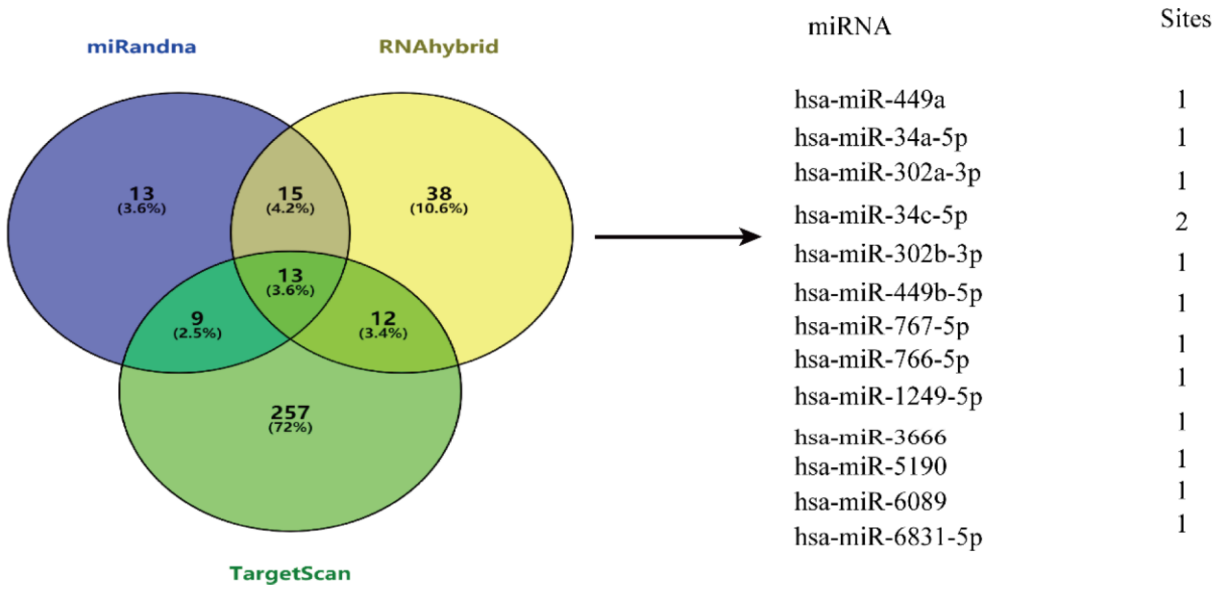

Supplementary Figure 2. Predicted miRNAs binding to CircCCNB1. The intersection of TargetScan, miRanda and RNAhybrid showed that CircCCNB1 acted as a sponge for 13 miRNAs. Among these, miR-449a were the top predicted miRNA targets of CircCCNB1.

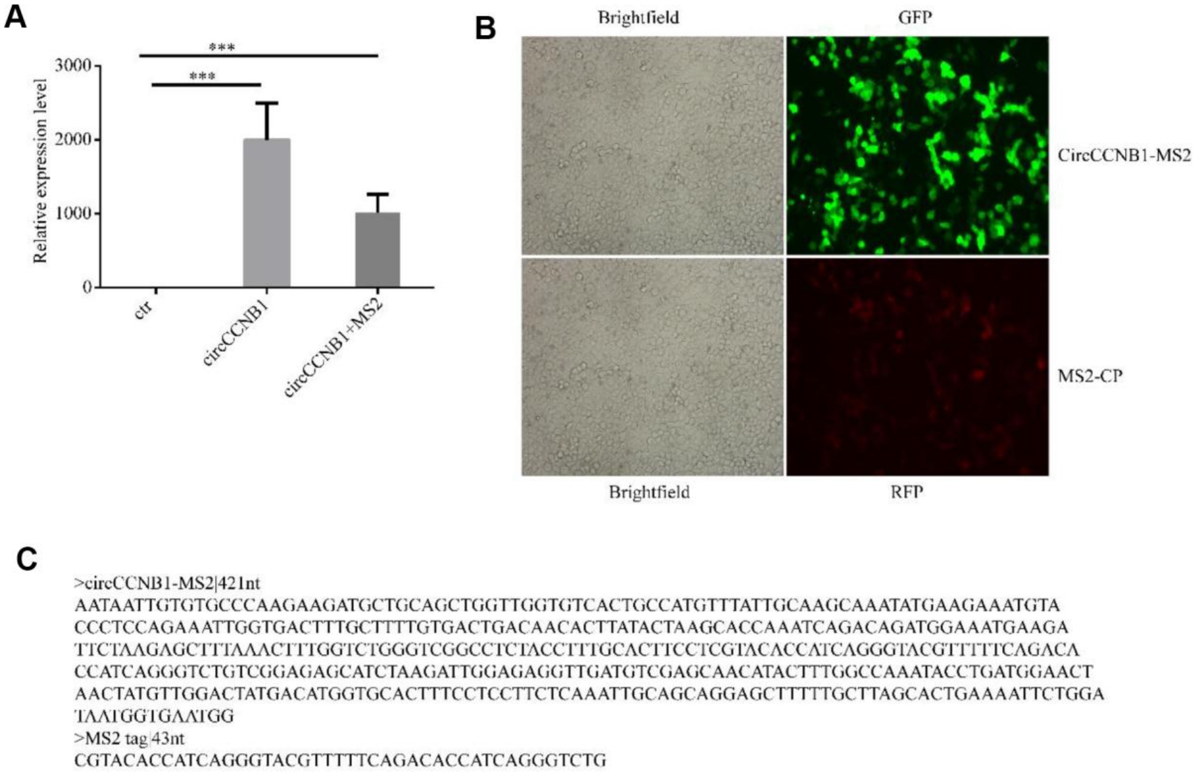

Supplementary Figure 3. Validating the expression efficacy of CircCCNB1-MS2 and MS2-CP in HEK293T. (**A**) Relative expression level in HEK293T cells after transfected with ctr, CircCCNB1, CircCCNB1-MS2, respectively, ***P<0.001. (**B**) expression efficacy of CircCCNB1-MS2 and MS2-CP in HEK293T cells (magnification, ×10). (**C**) Sequence of CircCCNB1-MS2 and MS2.
