# Supplementary material for: Circular RNA CircCCNB1 sponges micro RNA-449a to inhibit cellular senescence by targeting CCNE2
Source: Aging (Albany NY). 2019 Nov 25;11(22):10220–41. doi: 10.18632/aging.102449 (PMC6914408; doi:10.18632/aging.102449)
Supplement: Supplementary Tables [file aging-11-102449-s001..pdf]

SUPPLEMENTARY TABLES

Supplementary Table 1. Primers used in this study.

List of oligonucleotides sequences (F: Forward, R: Reverse)	5′→3′
**Primers for real-time PCR**	
CircNFKB1-Di-qF	GTGGACTACCTGGTGCCTCT
CircNFKB1-Di-qR	ATGAAACATTTGTTCAGGCCTTCC
CircUBXN7-Di-qF	CTATTCCGGCCACCCATTGA
CircUBXN7-Di-qR	AGGCCGTCGTCTTTTAGGAG
CircMIB1-Di-qF	GTTCTTTTGGAAGCTGGAGCAG
CircMIB1-Di-qR	TTGCTGGCGGCAGGTATC
CircPLEKHM1P1-Di-qF	GTCCAGACCATTGCGAGGAG
CircPLEKHM1P1-Di-qR	ACAAACGTCAGGTGCTCCAA
CircUBE2G1-Di-qF	CCCGCTGTGTAAGAAAAAGCC
CircUBE2G1-Di-qR	CCTGCAGAAAAGCCTTCCACT
CircCCNB1-Di-qF	GACATGGTGCACTTTCCTCC
CircCCNB1-Di-qR	ATGGCAGTGACACCAACCAG
CircGLIS3-Di-qF	CGCCCGCTATAAACTGCTGA
CircGLIS3-Di-qR	GCCAAAGACTCACGCGAAAT
CircDEK-Di-qF	GCCAGTGCTAACTTGGAAGAAGT
CircDEK-Di-qR	TCTTTTCCCTCTTGCCTTCCAC
CircBARD1-Di-qF	ATCGCTATTGCTGCTACCAGAG
CircBARD1-Di-qR	TCCTCCTAAACACACAGGCTC
CircNRDC-Di-qF	CCGTGAAGTTGAAGCTGTTGA
CircNRDC-Di-qR	AGGCATTATCACTACCCCCA
CCNB1-Di-qF	AATAAGGCGAAGATCAACATGGC
CCNB1-Di-qR	TTTGTTACCAATGTCCCCAAGAG
GAPDH-qF	ACAACAGCCTCAAGATCATCAGCAAT
GAPDH-qR	GTCCTTCCACGATACCAAAGTTGTCA
miR-449a-qF	CGTGGCAGTGTATTGTTAGCTGGT
miR-449a-RT	GTCGTATCCAGTGCAGGGTCCGAG GTATTCGCACTGGATACGACACCAGC
miR-449a-qR	GTGCAGGGTCCGAGGT
U6-F	AGAGAAGATTAGCATGGCCCCTG
U6-R	GTCGTATCCAGTGCAGGGTCCGAGGTATTCGCACTGGATACGACAAAAT ATG
**Primers for RT-PCR** (Co: convergence, Di: divergence)	
CircCCNB1-Co-F	TGCTTTTGTGACTGACAACACT
CircCCNB1-Co-R	ATGCTCTCCGAAGGAAGTGC
CircCCNB1- Di-F	ATGACATGGTGCACTTTCCTC
CircCCNB1- Di-R	ACCCTCCAGAAATTGGTGACT
CircDEK-Co-F	TCATCGTGGAAGGCAAGAGG
CircDEK-Co-R	AATGAGGACACAGTGCCTGG
CircDEK-Di-F	TGAAGAAACCCCCTACAGATGAA
CircDEK-Di-R	ACAAAGTTTCTGCCCCTTTCCT
CircBARD1-Di-F	GGAGCCTCCAGAAATGCTGTTA
CircBARD1-Di-R	TCCTTGTCACAGGACCCAATC
CircBARD1-Co-F	GAGCCTGTGTGTTTAGGAGGA
CircBARD1-Co-R	GGGGTGTAACACACTGGACA
CircNRDC-Di-F	TTGATAGATGGGCGCAGTTCTT
CircNRDC-Di-R	GACAGTGCGTTCACAATCAGTT

CircNRDC-Co-F	ATGGATTTGATGCCTTCCTGA
CircNRDC-Co-R	ACAGCTTCAACTTCACGGTCA
CircPLEKHM1P1-Di-F	ACTCCAAGAGAATGGCTCCAAG
CircPLEKHM1P1-Di-R	ACTCCATCAGGCCATTGTTCA
CircPLEKHM1P1-Co-F	TTGGAGCACCTGACGTTTGT
CircPLEKHM1P1-Co-R	CCTCCGTATCTTATGGCCCG
**Primers for circCCNB1 interference**	
sh-scramble	TTCTCCGAACGTGTCACGT
sh-circCCNB1-1	GGATAATGGTGAATGGAATAA
sh-circCCNB1-2	GAATGGAATAATTGTGTGCCC
**Primers for circCCNB1 overexpression**	
circCCNB1-F	cgGAATTCTAATACTTTCAGAATAATTGTGTGCCCAAGAAGATGCG
circCCNB1-R	cgGGATCCAGTTGTTCTTACCCATTCACCATTATCCAGAATTTTCAGTGC
**Primers for circCCNB1-MS2 construction**	
circCCNB1-MS2-F	cgGAATTCTAATACTTTCAGAATAATTGTGTGCCCAAGAAGA
circCCNB1-MS2-R	cgGGATCCAGTTGTTCTTACCCATTCACCATTATCCAGAATT

Supplementary Table 2. Top 5 dysregulated circRNAs in premature senescent 2BS cells.

Circ RNA ID	Log_2F^a	regulation	chromosome	circBase ID	Gene Name
hsa_circ_NFKB1	11.02	up	Chr4	hsa_circ_0008012	NFKB1
hsa_circ_UBXN7	12.34	up	Chr3	hsa_circ_0001380	UBXN7
has_circ_MIB1	11.02	up	Chr18	hsa_circ_0000836	MIB1
hsa_circ_PLEKHM1P1	11.82	up	Chr17	-	PLEKHM1P1
hsa_circ_UBE2G1	10.02	up	Chr17	hsa_circ_0004805	UBE2G1
hsa_circ_GLIS3	-11.02	down	Chr9	hsa_circ_0006370	GLIS3
hsa_circ_DEK	-11.17	down	Chr6	-	DEK
hsa_circ_CCNB1	-10.85	down	Chr5	hsa_circ_0001495	CCNB1
hsa_circ_BARD1	-8.24	down	Chr2	-	BARD1
hsa_circ_NRDC	-7.44	down	Chr1	-	NRDC

[a]FC fold change.
